# N6-methyladenosine methylation modification patterns reveal immune profiling in pancreatic adenocarcinoma

**DOI:** 10.1186/s12935-022-02614-x

**Published:** 2022-05-23

**Authors:** Hao Xu, Lu Yin, Qianhui Xu, Jingjing Xiang, Rujun Xu

**Affiliations:** 1 Department of Hepatobiliary and Pancreatic Surgery, The Second Affiliated Hospital, School of Medicine, Zhejiang University, Hangzhou, Zhejiang China; 2 Department of Pathology, Affiliated Hangzhou First People’s Hospital, Zhejiang University School of Medicine, Hangzhou, Zhejiang China

**Keywords:** Pancreatic adenocarcinoma, m6A modification patterns, Tumor immune microenvironment, Tumor mutation burden, TNFRSF21

## Abstract

**Background:**

Several studies have revealed that N6-methyladenosine (m6A) regulation is involved in various biological processes and cancer progression. Nevertheless, the potential effects of m6A modifications in the tumor immune microenvironment (TIME) and on immune regulation in pancreatic adenocarcinoma (PAAD) remains unclear.

**Methods:**

A consensus clustering algorithm was used to identify different m6A modification patterns and construct an m6A-associated gene signature based on 23 m6A regulators in PAAD. The CIBERSORT and ssGSEA algorithms were used to estimate the components of the immune cells in each sample. The PCA algorithm was used to develop the m6Ascore system for the evaluation of m6A modification patterns in each sample.

**Results:**

Two m6A modification patterns with different biological properties and prognoses were identified in 176 PAAD patient samples. The features of TIME between the two patterns were similar, with two definite immune phenotypes: immune-inflamed and immune-excluded. Based on the m6A phenotype-associated signature genes, we constructed an m6Ascore system to investigate the m6A modification pattern of each sample, profile the dissection of physiological processes, immune infiltration, clinical prognosis, immunotherapy, and genetic variation. Patients with low m6Ascore scores had better clinical outcomes, enhanced immune infiltration, and lower expression of immunotherapeutic drug targets, such as CD274 and PDCD1LG2. Further research indicated that the m6Ascore and tumor mutation burden were significantly correlated, and patients with low m6Ascore had higher mutation rates in SMAD4 and TTN. Moreover, TNFRSF21 was significantly upregulated in PAAD tumor tissues and cell lines. Lower expression of TNFRSF21 had a prominent advantage in survival and was correlated with a low level of immune infiltration. PAAD samples with different TNFRSF21 expression levels showed significantly distinct sensitivities to chemotherapeutic agents.

**Conclusions:**

This study revealed that m6A modification patterns could play an important role in the diversity and complexity of TIME, and the m6Ascore system could serve as an independent and powerful prognostic biomarker and is latently related to PAAD immunotherapies. Quantitative determination of m6A modification patterns in individual patients will be instrumental in mapping the TIME landscape and further optimizing precision immunotherapy.

**Supplementary Information:**

The online version contains supplementary material available at 10.1186/s12935-022-02614-x.

## Introduction

N6-methyladenosine (m6A), the most prevalent epigenetic internal modification of eukaryotic messenger RNAs (mRNAs) and noncoding RNAs, is a methylation occurring in the N6-position of adenosine [1]. M6A modification is controlled by many m6A regulator types. For instance, m6A is installed via m6A methyltransferases, which are also called “writers,” and removed by m6A demethylases, which are recognized as “erasers,” then termed reader proteins (“readers”) [2, 3]. During physiological processes and disease progression, m6A regulates RNA metabolism, including translation, splicing, export, and degradation [4, 5]. Accumulating evidence has shown that m6A regulators are involved in cancer progression and immunomodulatory abnormalities through abnormal mutations and expression, which affect biological processes [6, 7]. A thorough understanding of the expression and genetic alterations of m6A will benefit the recognition of m6A-based therapeutic targets to further predict prognosis and improve clinical outcomes [8].

Pancreatic adenocarcinoma (PAAD) is among the most refractory cancers worldwide, with a high cancer-related mortality rate and poor prognosis [9, 10]. PAAD is highly malignant and prone to metastasis, and the resectability rate of PAAD patients is low. Therefore, efficient treatment methods for PAAD remain a challenge [11–13]. An increasing number of studies have demonstrated the importance of the complexity and diversity of the tumor immune microenvironment (TIME) [14, 15]. The intercellular relationships among tumor cell subsets, immune cells, and involved signaling pathways play important roles in the occurrence and progression of tumors [16, 17]. Tumor-infiltrating and circulating CD4 + cytotoxic T lymphocytes (defined as CD4 + T cells with direct lytic activity via expression of perforin and granzyme) have been proven independent prognostic markers of overall survival in many tumors types [18]. Remarkably, molecular-targeted therapies and immune checkpoint inhibitors have shown limited efficacy against PAAD. Most PAAD patients do not benefit from therapy [13]. Thus, the investigation of tumor immune phenotypes based on TIME characterization contributes to the prediction of immunotherapeutic outcomes and benefits for the discovery of immunotherapeutic molecular targets for PAAD [19].

Recently, increasing research has revealed that m6A modifications and immunological regulation are closely related. For instance, *FTO* upregulates *Sox10, PD-1*, and *CXCR4* by inhibiting YTHDF2-mediated degradation and suppressing the response to immunotherapy with PD-1 blockade, thereby damaging the IFN γ-induced cytotoxicity of melanoma cells [20]. YTHDF2 bound m6A-modified circRNA to inhibit circRNA immunity, results in the neglect of “self circRNA” and RIG-I inactivation [21]. YTHDF1 suppresses dendritic cells presenting neoantigens to T lymphocytes and promotes tumor cell immune escape by promoting lysosomal-related degradation of neoantigens [22]. Therefore, recognizing the characteristics of multiple m6A regulator-mediated TIME will aid the prognosis estimation and individualized clinical intervention to further explore precision immunotherapy.

This study analyzed the genomic and transcriptomic data of PAAD samples from TCGA-PAAD project datasets to explore the potential relationship between m6A modification patterns and the TIME landscape. Consensus clustering determined two different m6A modification patterns, and the features of TIME in the two pattern subtypes could be termed as two known immune phenotypes. Moreover, we established an m6A-based scoring system to estimate m6A modification patterns and the prognosis of individual samples. We further investigated the potential roles of the m6Ascore in clinicopathological variables and assessed its relationship with immunotherapeutic response. The correlation between the m6Ascore and tumor mutational burden (TMB) was verified. Finally, the biological functions of TNF receptor superfamily member 21 (TNFRSF21) in prognostic prediction, immune infiltration, and chemotherapy were further explored to provide robust insights into clinical therapeutic strategies for PAAD. These studies revealed that m6A modification had a significant impact on the formation of various TIME and assisted personalized immunotherapeutic strategies in PAAD.

## Methods

### Public datasets collection and preprocessing

Gene expression annotation and clinical data were downloaded from the Cancer Genome Atlas database (TCGA, https://cancergenome.nih.gov/). Totally 176 PAAD samples from TCGA-PAAD were used for further analysis. TCGA RNA sequencing information (FPKM format) of gene expression was obtained from the Genomic Data Commons (GDC, https://portal.gdc.cancer.gov/) and transformed into transcripts per kilo base million value. To remove the batch effects from non-biological technical biases, the “sav” R package with the ComBat algorithm was performed. The genomic mutation profiles, including simple nucleotide variation and copy number variation (CNV) of TCGA-PAAD cohort, were curated from the GDC. The CNV landscape of 23 m6A regulators in human chromosomes was plotted using “Rcircos” R package. Non-synonymous mutation (including frameshift, inflame, missense, nonsense, and splice site mutations) counts were recognized as TMB. The expression levels of the m6A regulators from the TCGA-PAAD project are listed in Additional file 7: Table S1.

### Consensus clustering based on 23 m6A regulators

According to the existing research on m6A modification, 23 known m6A methylation modification regulators have been utilized to uncover m6A methylation modification patterns [23–26]. These m6A regulators comprise eight “writers” (KIAA1429, CBLL1, METTL3, METTL14, RBM15, WTAP, RBM15B, and ZC3H13), two “erasers” (FTO and ALKBH5), and 13 “readers” (FMR1, ELAVL1, HNRNPC, HNRNPA2B1, IGF2BP1, IGF2BP2, IGF2BP3, LRPPRC, YTHDC1, YTHDC2, YTHDF1, YTHDF2, and YTHDF3). An unsupervised clustering algorithm was used to determine different m6A modification patterns based on 23 m6A regulators to partition the PAAD subjects. R package “ConsensuClusterPlus” was employed to implement these analyses, and repetitions of 1000 times were performed for guaranteeing the stability of clustering.

### Gene set variation analysis (GSVA) and gene ontology (GO) enrichment analysis

GSVA [26] with the 'GSVA' R package was used to explore the variation in biological characterizations of distinct m6A modification patterns. The acknowledged biological processes were derived from the gene sets of “h.all.v7.4.symbols.gmt [Curated]’ (downloaded from the Molecular Signatures Database). The Entrez ID for each gene was obtained using the R package “org.Hs.eg.db”. The GO annotation for m6A phenotype-related genes was analyzed and visualized with R packages “clusterProfiler,” “enrichplot” and “ggplot2”.

### Estimation of tumor immune microenvironment contexture

Single-sample gene set enrichment analysis (ssGSEA) was used to estimate the relative level of immune infiltration. The relative abundance of each leukocyte subset was represented by an enrichment score in ssGSEA and normalized to a unity distribution from 0 to 1. The biosimilarity of infiltrating immune cells was estimated using multidimensional scaling and a Gaussian fitting model. The deconvolution approach, CIBERSORT (http://cibersort.stanford.edu/), was employed to quantify the components of 22 distinct immune cell types based on gene expression profiling. To uncover the potential role of m6A score in immune infiltration, seven algorithms (XCELL, TIMER, QUANTISEQ, MCPcounter, EPIC, CIBERSORT, and CIBERSORT-ABS) were used. Spearman correlation analysis was performed to investigate the correlation between the risk score and immune infiltration.

### Calculation of tumor mutational burden

TMB was defined as the number of somatic, coding, base replacement, and insert-deletion mutations per mega base of the genome examined, using non-synonymous and code-shifting indels under a 5% detection limit. TMB scores for each sample were calculated by dividing the number of somatic mutations by the total exon length. The “maftools” R package [27] was employed to compute the total number of somatic non-synonymous point mutations within individual subjects.

### Identification of m6A modification phenotype-related differentially expressed genes (DEGs)

Based on the results of the consensus clustering algorithm, the samples were partitioned into three distinct m6A modification phenotypes. Next, m6A modification-correlated DEGs were identified among these m6A modification patterns by employing R package 'limma'. DEGs with an adjusted P-value < 0.001 were considered significant and were employed for further analysis.

### Development of the m6A scoring scheme (m6Ascore)

An m6A scoring system was developed using the principal component analysis (PCA) to evaluate m6A modification patterns in each sample. The patients were clustered into different subtypes using an unsupervised clustering algorithm to analyze overlapping DEGs identified from previous distinct m6A clusters. Prognostic analysis was performed for each gene using univariate Cox regression analysis. Genes with prognostic significance were further analyzed. Then, the expression profiles of the final identified genes were extracted, and the PCA was used to develop m6A relevant gene signatures. Principal components 1 and 2 were curated to serve as the m6A signature (m6sig) score.

The superiority of this algorithm is that it focuses on the score of the set with the largest block of well-correlated (or inverse-correlated) genes in the set, while down-weighting contributions from genes that do not track with other set members. A formula similar to that used in previous studies was adopted to quantify the m6Ascore [28, 29]. The formula is as follows.

$${\text{m6Ascore}}= \sum ({\text{PC1i}}\, + \,{\text{PC2i}})$$

where i is the expression of m6A phenotype-related genes.

### Cell culture and samples

We further evaluated the *TNFRSF21* expression in PAAD and normal pancreatic cell lines. HPNE and PANC-1 cells were cultured in DMEM (Biological Industries) containing 10% fetal bovine serum (Biological Industries), whereas BxPC-3 cells were cultured in RPMI-1640 (Biological Industries) supplemented with 10% fetal bovine serum. For the assessment of TNFRSF21 in cells, we extracted the total RNA using an RNA-Quick purification kit according to the manufacturer’s instructions (Vazyme, RN001). The PrimeScriptTM RT Reagent Kit with gDNA Eraser (Takara, RR047A) was used to reverse transcribe the total RNA into cDNA. Quantitative real-time polymerase chain reaction (qPCR) was conducted in an Applied Biosystems 7500 Fast Real-Time PCR System using TB Green Premix EX Taq™ II (Takara, RB820A). The expressions of TNFRSF21 were analyzed using the 2-ΔΔC method, and β-actin was used as an endogenous control. The primer sequences used for PCR are listed in Additional file 7: Table S7.

### Prediction of chemotherapeutic effect

To estimate the sensitivity of chemotherapy, the R package pRRophetic was used to estimate the half-maximal inhibitory concentration (IC50) of PAAD samples in different groups. By constructing a ridge regression model based on the Genomics of Drug Sensitivity in Cancer (www.cancerrxgene.org/) cell line expression spectrum and TCGA gene expression profiles, the pRRophetic package could estimate the IC50 of chemotherapeutic drugs.

### Prediction of patients’ response to immunotherapy

To further explore the potential role of *TNFRSF21* in immunotherapeutic prediction, the immunophenoscore (IPS), which quantifies the determinants of tumor immunogenicity and characterizes the cancer antigenomes and intratumoral immune landscapes, was used as a novel and robust predictor of response to immunotherapeutic regimens,. A scoring system was constructed based on a panel of immune-related genes from the four classes: suppressor cells, effector cells, immunomodulators, and MHC-related molecules. The weighted averaged Z score was computed by averaging the sample-wise Z scores of the five classes within the respective category, and the sum of the weighted averaged Z scores was termed as the IPS.

## Statistical analyses

Statistical analyses in this study were performed using R-4.0.3. For quantitative data, statistical significance for normally distributed variables was estimated using Student's t-tests, and non-normally distributed variables were analyzed using the Wilcoxon rank-sum test. For comparisons of more than two groups, the Kruskal–Wallis test and one-way analysis of variance were used as nonparametric and parametric methods, respectively. Kaplan–Meier survival analysis and the Cox proportional hazards model were applied to analyze the correlation between m6A modification patterns and overall survival (OS) time using the ‘Survminer' R package (0.4.6). The surv-cutpoint function from the R package 'survival' was used to partition the PAAD samples into high/low m6Ascore subgroups. Participants with detailed clinical data were included and adjusted for confounding factors in the multivariate regression model. All comparisons were two-sided with an alpha level of 0.05, and the Benjamini–Hochberg method was applied to control the false discovery rate (FDR) for multiple hypothesis testing.

## Results

### Genetic variation of m6A RNA methylation regulators in PAAD

**Fig. 1:**
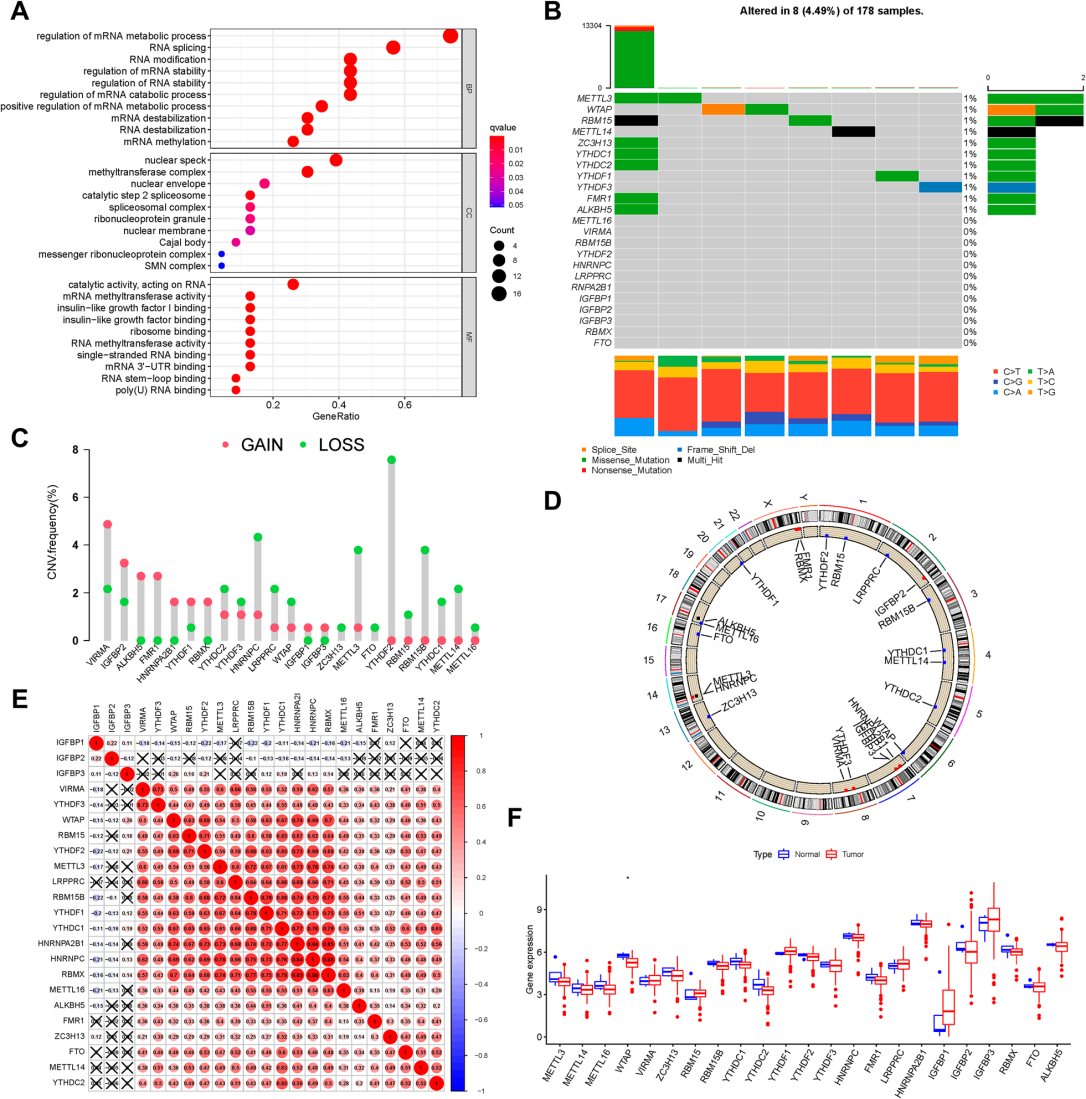
The landscape of genetic alterations of m6A regulators in PAAD. **A** GO enrichment analysis of 23 m6A regulators. The x-axis indicates the gene ratio for each GO term. **B** Eight of the 178 PAAD patients experienced genetic alterations in 23 m6A regulators, with a frequency of 4.49%, mostly including missense mutations, multi-hit, and deep deletions. The numbers on the right indicate the mutation frequency of each regulator. Each column represents the individual patient. **C** The CNV mutation frequency of 23 m6A regulators was prevalent. The column represents the frequency of the alterations. Deletion frequency, green dot; amplification frequency, red dot. **D** Location of CNV alterations in m6A regulators on chromosomes. **E** Broad co-expression correlations among the 23 m6A RNA modification regulators in PAAD. “X” means P ≥ 0.05. **F** Difference in mRNA expression levels of 23 m6A regulators between normal and PAAD samples. Asterisks (*) represent the statistical P-values (*P < 0.05, **P < 0.01, and ***P < 0.001).

In this study, the roles of 23 m6A modification regulators (“readers”: IGF2BP1, IGF2BP2, IGF2BP3, YTHDC1, YTHDC2, YTHDF1, YTHDF2, YTHDF3 ELAVL1, FMR1, HNRNPA2B1, HNRNPC, and LRPPRC; “erasers”: ALKBH5 and FTO; and “writers”: CBLL1, KIAA1429, METTL3, METTL14, RBM15, RBM15B, WTAP, and ZC3H13) were investigated in pancreatic cancer. GO annotation analyses of 23 m6A regulators was performed, and significant enrichment of the biological pathways was observed (Fig. 1A). We discovered that 8/178 (4.49%) samples possessed somatic mutations of m6A regulators through the delineation of mutation profiles of 23 m6A regulators in TCGA-PAAD samples. These mutations include: missense mutations, multi-hit and deep deletions (Fig. 1B). We identified mutated genes in PAAD with ranked percentages, including *METTL3* (1%), *WTAP* (1%), *RBM15* (1%), *METTL14* (1%), *ZC3H13* (1%), *YTHDC1*(1%), *YTHDC2* (1%), *YTHDF*1 (1%), *YTHDF3*(1%), *FMR1* (1%), and *ALKBH5(*1%). The prevalence of CNV mutations in the 23 m6A regulators was further analyzed, revealing that *VIRMA, IGFBP2, ALKBH5, FMR1*, and *HNRNPA2B1* experienced widespread CNV amplification, whereas *HNRNPC, YTHDF2, METTL3, RBM15B,* and *YTHDC1/2/3* possessed general CNV deletions (Fig. 1C). The CNV mutation locations of the 23 m6A regulators in the chromosomes are shown in Fig. 1D. Spearman’s correlation was analyzed to explain the mutual connection among the 23 m6A regulators (Fig. 1E). The results revealed that the “readers” *IGF2BP1* and *IGF2BP2*, experienced a prominent negative relationship with other m6A regulators, but the other 21 m6A regulators had positive correlations with each other. Subsequently, we explored the difference in expression between tumor tissues and adjacent tissues of PAAD and observed that *WTAP* was notably downregulated in tumor tissues, whereas *RBM15*, *IGFBP1/3*, and *YTHDF1* seemed upregulated in tumor samples (Fig. 1F). Kaplan–Meier survival analysis of the low and high expression of these m6A regulators indicated that 13/23 m6A regulators were significantly associated with survival. The overexpression of *ALKBH5, FTO, IGFBP2, METTL3,* and *METTL16* exhibited a prominent advantage in survival, whereas the low expression of *FMR1, HNRNPC, IGFBP3, LRPPRC, RMB15, VIRMA, YTHDF3,* and *ZC3H13* was associated with a better prognosis (Additional file 1: Fig. S1). The above research supports the remarkable distinctions and intrinsic relations in transcriptomic and genomic maps of m6A modification regulators between tumor and normal tissues of PAAD. Thus, the variation in the expression and genetic alterations of m6A regulators might provide novel insights into the occurrence and development of PAAD.

### Construction of m6A methylation modification patterns

**Fig. 2:**
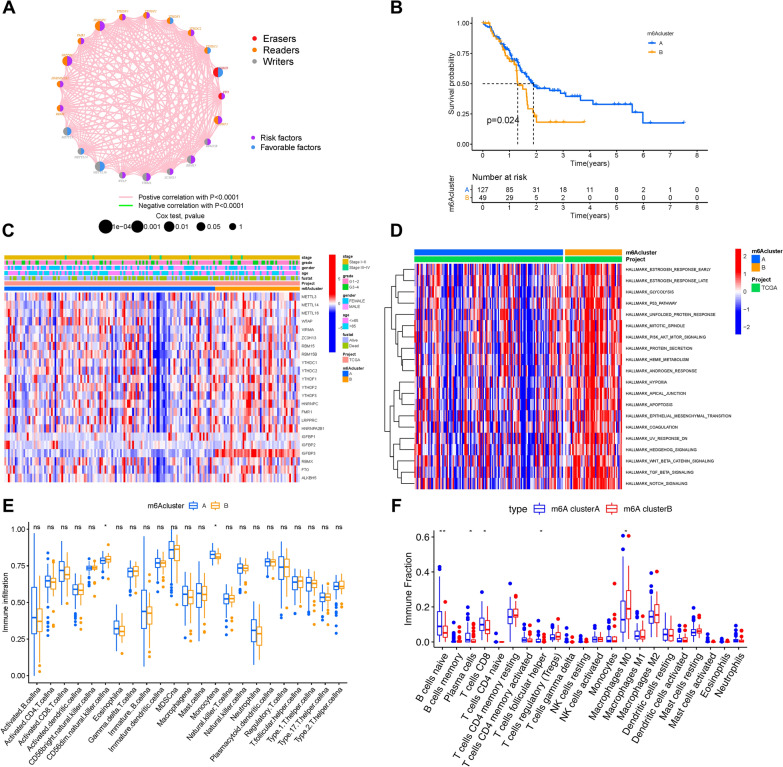
Clustering of m6A methylation modification patterns and the biological characteristics and TIME contexture. **A** The interaction of expression between 23 m6A regulators in PAAD. The m6A regulators in two RNA modification types are depicted by circles in different colors. “Readers”, orange; “Writers”, grey; “Erasers”, red. The lines connecting m6A regulators represent their interaction with each other, and thickness shows the correlation strength between regulators. Negative correlation is marked with green and positive correlation with pink. The size of each circle represents the prognosis effect of each regulator scaled by P-value, and the range of values calculated by Log-rank test are P < 0.001, P< 0.01, P < 0.05 and P < 0.1, respectively. Protective factors for patients' survival are represented by blue and risk factors are represented by purple. **B** Survival analyses for the two m6A modification patterns based on 176 patients including 127 cases in m6Acluster A, 49 cases in m6Acluster B. (P = 0.024, Log-rank test). **C** Unsupervised clustering of 23 m6A regulators in the combined cohort. The m6A cluster, survival status, gender, tumor stage, and age were used as patient annotations. Red represents the high expression of regulators and blue represents low expression. **D** GSVA enrichment analysis showing the activation states of biological pathways in m6Acluster A and m6Acluster B modification patterns. The heatmap was used to visualize these biological processes, and red represents activated pathways and blue represents inhibited pathways. **E** ssGSEA algorithm; **F** CIBERSORT approach. The fraction of tumor-infiltrating lymphocyte cells in two m6A modification patterns. Within each group, the scattered dots represent TIME cell expression values. The thick line represents the median value. The bottom and top of the boxes are the 25th and 75th percentiles (interquartile range). The whiskers encompass 1.5 times the interquartile range. The statistical difference of two gene clusters was compared through the Kruskal–Wallis H test. *P < 0.05, **P < 0.01, and ***P < 0.001

The PAAD dataset with the corresponding clinical data was included in the combined cohort. The m6A regulator network showed a comprehensive view of 23 m6A regulator connections and their corresponding prognostic value (Fig. 2A), from which we found that m6A regulators with the same biological function experienced significant correlation, which was similar to the cross-talk among “readers”, “writers”, and “erasers”. Therefore, the correlation among m6A regulators plays an important role in the development and prognosis of PAAD. The “ConsensusClusterPlus” R package was employed to stratify samples into two m6A modification patterns based on the expression of 23 m6A regulators (Additional file 2: Fig. S2A–D). Then, two different modification patterns, pattern A (127 samples) and pattern B (49 samples), were finally determined using unsupervised clustering. The patterns were designated as m6Acluster A and m6Acluster B. Kaplan–Meier survival analysis of the two m6A modification clusters indicated that m6Acluster A experienced a notable advantage in survival, while m6Acluster B had a poor clinical outcome (Fig. 2B). The heatmap shows the differential expression of 23 m6A regulators based on the classification of the m6A cluster, survival status, gender, tumor stage, and age (Fig. 2C). The two m6A modification patterns exhibited different expression levels of m6A regulators, and it seemed like almost all m6A regulators were upregulated in the m6Acluster B subtype (Fig. 2C).

### The m6A modification patterns characterized with different immune phenotypes

GSVA against the hallmark gene set was applied to further investigate the biological behaviors of the two m6A modification patterns (Fig. 2D). GSVA presented that m6Acluster B possessed enrichment pathways related to stromal pathways and carcinogenic activation, such as TGF-β, Wnt-β-catenin, Notch, PI3K-AKT-mTOR signaling, and hedgehog signaling pathways.

Furthermore, m6Acluster B was also significantly enriched in both protein secretion and glycolysis. Subsequently, the relative subpopulations of 23 infiltrating immune cell abundances were confirmed by comparing the two m6A modification patterns using ssGSEA and CIBERSORT (Fig. 2E, F). Inflammatory-response cell subpopulations that participate in anti-tumor processes, such as naive B lymphocytes, CD8 + T lymphocytes, and plasma cells, were markedly up-regulated in the m6Acluster A. We discovered that M0 macrophages were mainly rich in m6Acluster B. Although there were no significant differences, M1 macrophages experienced a higher trend in the m6Acluster A, whereas M2 macrophages presented more fraction in the m6Acluster B. Taken together, these two m6A modification patterns were characterized by distinct profiles of infiltrating immune cells. A large quantity of tumor-infiltrating lymphocytes, such as CD8 + T lymphocytes and plasma cells, are markedly correlated with anti-tumor immune status [30, 31]. Thus, m6Acluster A could be considered an immune-inflamed phenotype with abundant activated lymphocytes. Due to the retention of stroma peripheral cancer nests, numerous infiltrating immune cells could not penetrate the tumor parenchyma in tumors of the immune-excluded phenotype [32]. We inferred that overexpression of stromal elements would weaken the efficacy of anti-tumor immunotherapy in the m6Acluster B. In general, m6Acluster A was characterized by abundant infiltration of immune cells and immune activation, identified as the immune-inflamed phenotype. However, m6Acluster B was characterized by infiltrating innate immune cells, and it exhibited abundant pathways markedly related to stromal pathways and carcinogenic activation. Therefore, m6Acluster B is considered an immune-excluded phenotype.

### Identification of m6A phenotype-related DEGs in PAAD

**Fig. 3:**
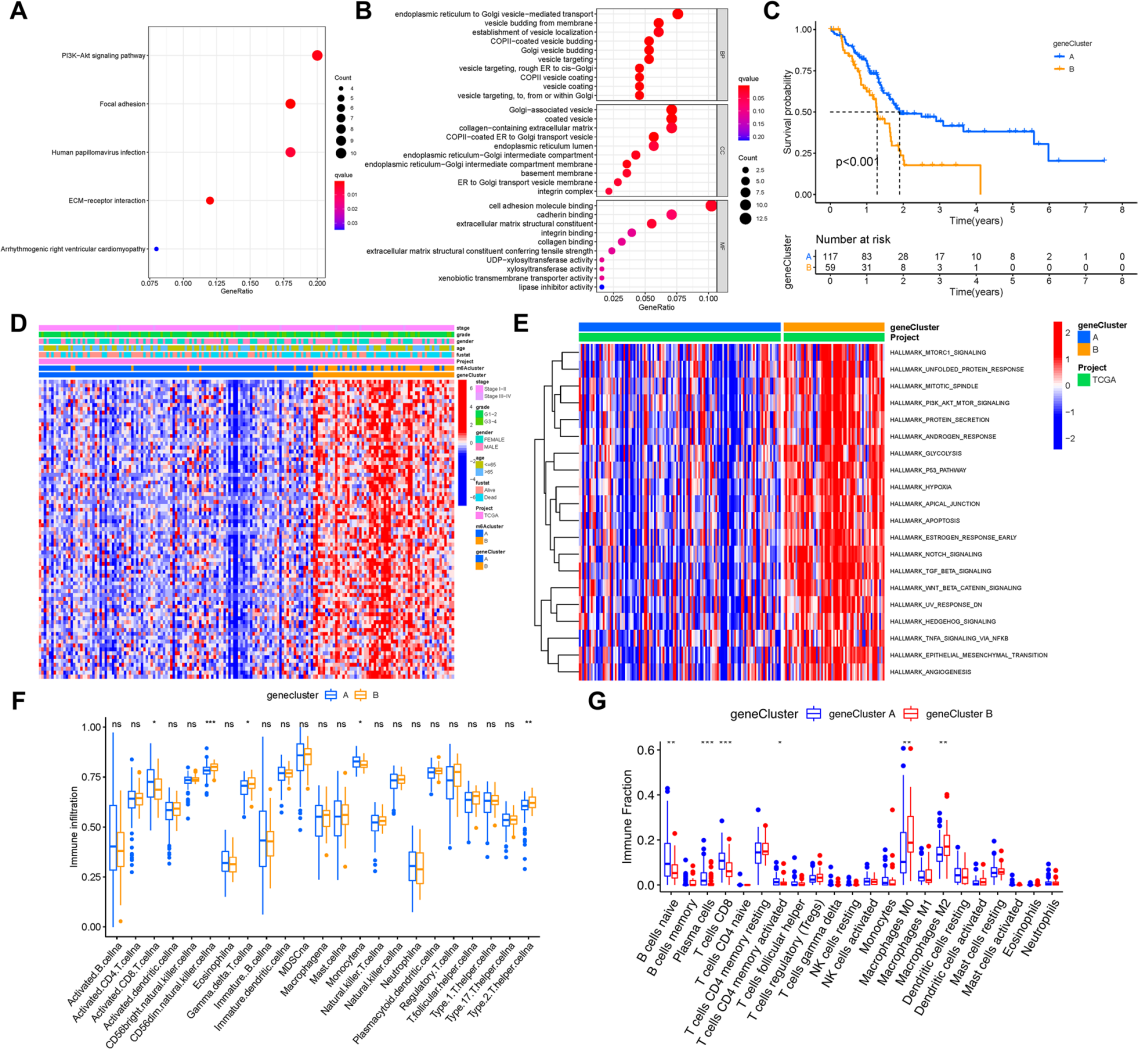
Construction of differential expression of m6A gene signatures and functional annotation. **A, B** Functional annotation for m6A-related genes using GO enrichment analysis. The color depth of the bar plots represents the number of genes enriched. **C** The survival curves of the m6A phenotype-related gene signatures were estimated by the Kaplan–Meier plotter. (P < 0.001, Log-rank test). **D** Unsupervised clustering of overlapping m6A phenotype-related DEGs to classify patients into different genomic subtypes, termed as m6A gene Cluster A and B, respectively. The gene signature subtypes, m6A clusters, survival status, tumor stage, gender, and age were used as patient annotations. **E** GSVA enrichment analysis showing the activation states of biological pathways in m6A gene Cluster A and gene Cluster B modification patterns. The fraction of tumor-infiltrating lymphocytes in three m6A modification patterns. Within each group, the scattered dots represent TIME cell expression values. **F** ssGSEA algorithm; **G** CIBERSORT approach

The Limma package was used to determine 169 DEGs, which were regarded as the pivotal distinct index of two m6A modification patterns, to further explore the latent alterations of m6A-associated transcriptional expression between two m6A modification phenotypes (Additional file 3: Fig. S3A). Figure 3A, B shows the biological processes in the GO enrichment analysis of DEGs. GO enrichment pathway analysis revealed that DEGs were mainly enriched in endoplasmic reticulum to golgi vesicle-mediated transport, vesicle budding from membrane and establishment of vesicle budding in biological processes; golgi-associated vesicle, coated vesicle and collagen-containing extracellular matrix in cellular components; cell adhesion molecule binding, cadherin binding and extracellular matrix structural constituent in molecular function. Additionally, GO enrichment pathway analysis indicated that the DEGs were closely related to the PI3K-Akt signaling pathway, focal adhesion, and human papillomavirus infection.

The 169 most representative m6A phenotype-related genes were analyzed by unsupervised clustering analyses, and the samples were divided into different transcriptomic phenotypes, from which we could better investigate the underlying molecular mechanisms (Additional file 4: Fig. S4A–D). These stratifications classified the samples into two m6A gene signature subgroups with different clinical variables, which were named m6A gene clusters A and B.

Kaplan–Meier survival analysis of two m6A gene signature subgroups indicated that gene cluster A, which contained 117 patients, exhibited an absolute advantage in overall survival. In contrast, 59 patients in gene cluster B had poorer prognosis (Fig. 3C). A heatmap was used to visualize the distinct clinicopathologic features and transcriptomic variation between the two m6A gene signature subgroups (Fig. 3D).

GSVA against the hallmark gene set was performed to further explore the biological behaviors between the two m6A gene subgroups (Fig. 3E). The results of GSVA revealed that m6A gene cluster B presented enrichment pathways prominently associated with almost all biological processes, including mitotic spindle, protein secretion, glycolysis, and apoptosis, as well as stromal pathways and carcinogenic activation, such as the TGF-β, Wnt-β-catenin, Notch, p53 pathway, and PI3K-AKT-mTOR signaling pathways. To explore the latent impact of m6A modification patterns on TIME, CIBERSORT and ssGSEA algorithms were further performed to assess the relative subpopulations of immune infiltration. The results showed that m6A gene cluster A was significantly enriched with naive B cells, plasma cells, and activated CD8 + T cells, which were abundant immune cells (including humoral immunity and cellular immunity), while m6A gene cluster B was remarkably rich in infiltration of M0 and M2 macrophages (Fig. 3F, G).

All of these analyses implied that m6A methylation modification had an indispensable impact on the regulation of shaping TIME landscapes.

### Construction of the m6Ascore

We revealed the potential roles of m6A modification or gene patterns in prognostic prediction and information on TIME. Nevertheless, the above studies only focused on the sample population of the database and might not be precisely applied to individuals. Therefore, on account of the complexity and heterogeneity of m6A modification in tumors, we constructed a scoring system, named as the m6Ascore, which is based on two m6A phenotype-associated signature genes, to explore the m6A modification pattern of individual PAAD patients.

**Fig. 4:**
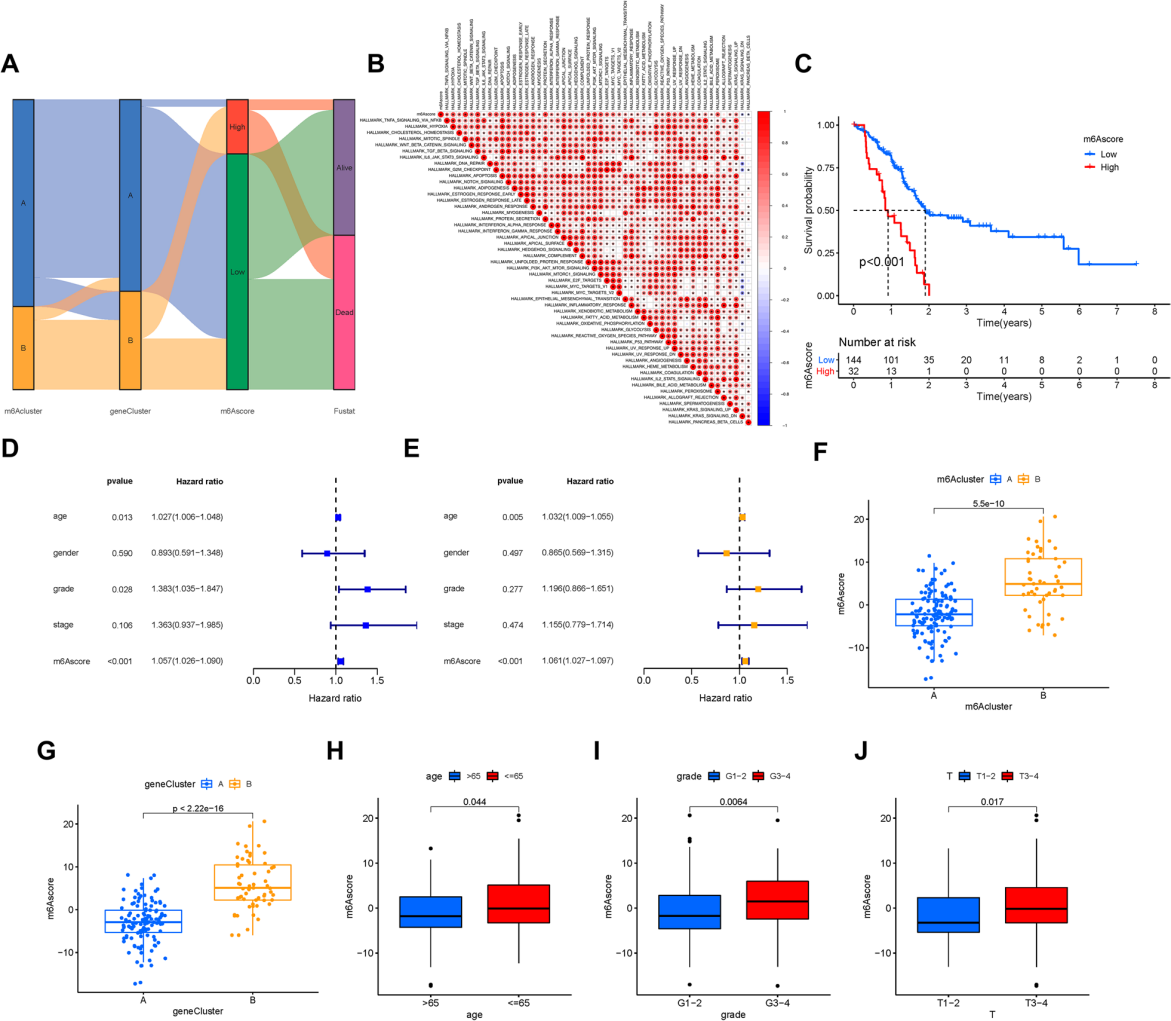
Construction of m6Sig score and explore the relevance of clinical features. **A** Alluvial diagram of m6A clusters in groups with different m6A-gene clusters, m6Ascores and survival status. **B** Correlations between m6Ascore and the well-established biological gene signatures using Spearman analysis. The negative correlation is marked with blue and positive correlation with red. **C** Kaplan–Meier curves for high and low m6Ascore patient groups. Log-rank test, P < 0.001. **D** Univariate Cox regression results of overall survival. The length of the horizontal line represents the 95% confidence interval for each group. The vertical dotted line represents the hazard ratio (HR) of all patients. **E** Multivariate Cox regression results of overall survival. The length of the horizontal line represents the 95% confidence interval for each group. The vertical dotted line represents the hazard ratio (HR) of all patients. **F** The difference of m6Ascore among distinct m6A gene clusters, P < 0.001. **G** The difference of m6Ascore among distinct m6A gene clusters, P < 0.001. Clinical significance of the m6Ascore. **H** Age. **I** Grade. **J** Tumor

Individual changes in patient attributes are summarized in an alluvial diagram (Fig. 4A). We analyzed the correlation between the m6Ascore with the well-established signatures. A heatmap of the correlation matrix shows the biological function of the m6Ascore. The m6Ascore was significantly positively correlated with most biological processes, such as the TGF-β, Wnt-β-catenin, PI3K-AKT-mTOR, and Notch signaling pathways (Fig. 4B). Moreover, the patients were classified into subgroups of high- or low-m6Ascore to reveal the prognostic value of the m6Ascore system in predicting overall survival time. The low-m6Ascore was associated with a better prognosis in the combined cohort (Fig. 4C).

Univariate (hazard ratio (HR) 1.057(1.026–1.090), P < 0.001, Fig. 4D) and multivariate (HR 1.061(1.027–1.097), P < 0.001, Fig. 4E) Cox regression analyses were performed to explore the role of the m6Ascore in forecasting the prognosis of PAAD patients, which demonstrated that the m6Ascore was an independent and powerful prognostic biomarker for predicting prognosis. These analyses strongly implied that the m6Ascore system could reflect the features of m6A modification patterns and predict prognosis in PAAD. Moreover, we explored the difference of m6Ascore in m6A methylation modification patterns and m6A gene signature subgroups. Notably, patients in m6Acluster B and gene cluster B had higher m6Ascore (Fig. 4F, G).

### Correlation of the m6Ascore with clinical traits

The potential role of m6Ascore in clinicopathological variables was further investigated. The subgroup with ≤ 65 had obviously higher m6Ascore (P < 0.05, Fig. 4H). The higher m6Ascores were concentrated in grade 3–4 (P < 0.05, Fig. 4I) and T3-4 (P < 0.05, Fig. 4J), while lower m6Ascores were accumulated in the subgroup of > 65, grade 1–2 and T1-2. However, the m6Ascore was not significant among the subgroups of the clinical stage (P > 0.05, Additional file 3: Fig. S3B).

Based on different clinical features, we divided PAAD patients into distinct subgroups and further explored whether the risk score could identify differences in the prognosis of PAAD patients using stratification analysis. When samples were divided according to age, the m6Ascore was still predictive of prognosis, and a lower m6Ascore was significantly correlated with a better outcome (Additional file 5: Fig. S5A, B). Similarly, m6Ascore acted as an excellent prognostic marker for patients based on sex (Additional file 5: Fig. S5C, D), early or late grade cancer (Additional file 5: Fig. S5E, F), and stage I-II cancer (Additional file 5: Fig. S5G, H). These results indicate that the m6Ascore system is closely related to clinical features. Therefore, the m6Ascore system could serve as a useful prognostic predictor independent of other clinical traits.

### Correlation of risk signature with immune cells infiltration

**Fig. 5:**
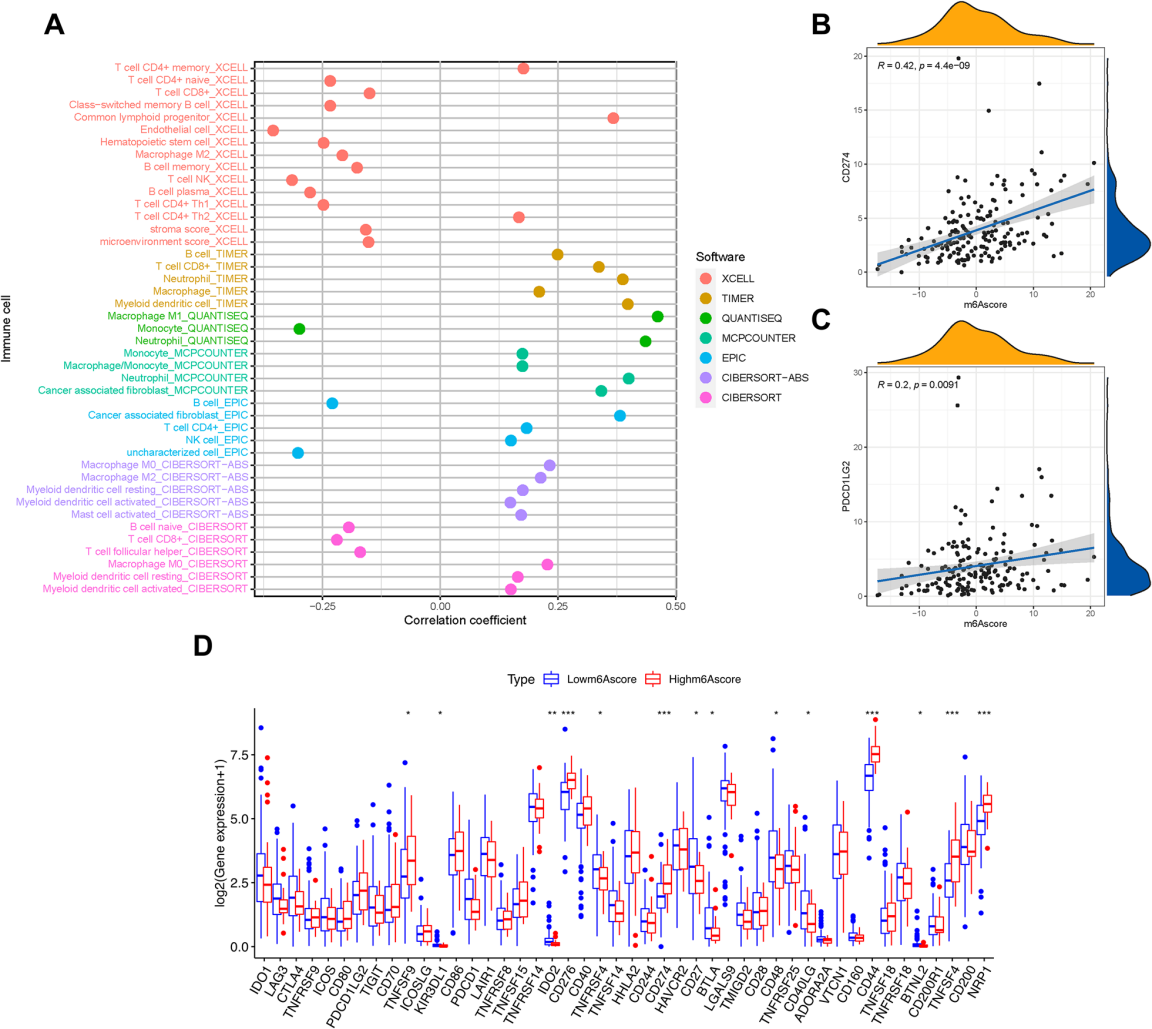
Estimation of tumor-infiltrating cells and expression level of immunotherapeutic hub genes. **A** The difference of tumor-infiltrating cells between patients in the low-m6Ascore and high-m6Ascore groups, as shown by Spearman correlation analysis. **B** Correlation between prognostic m6Ascore and CD274, **C** Correlation between prognostic m6Ascore and PDCD1LG2. **D** Expression level of immune checkpoint blockade genes between two risk-groups

Based on previous research, we revealed that m6A modification patterns and immune cell infiltration are closely related. Next, we explored the potential role of the m6Ascore in terms of the complexity and diversity of the TIME context. The results illustrated that the m6Ascore was negatively correlated with the subpopulations of B lymphocytes and CD8 + T lymphocytes, whereas it was positively correlated with myeloid dendritic cells, M0 macrophages, M2 macrophages, and cancer-associated fibroblasts (Fig. 5A). The results of Spearman’s correlation analysis are shown in detail in Additional file 7: Table S6. The results indicated that the m6Ascore had an indispensable impact on the immunological cross-talk of the TIME.

### Immunotherapeutic benefits of m6Ascore

Immunotherapy using anti-PD-1/CTLA-4 drugs has achieved encouraging success in clinical anti-cancer treatment regimens.. The immunotherapeutic hub targets, such as CD274, PDCD1LG2, CTLA‐4, etc. [33–35], are strongly associated with the clinical outcome of immunotherapy. Correlation of the m6Ascore with immunotherapy targets was further analyzed. It was observed that the m6Ascore was positively and significantly correlated with CD274 (r = 0.42; P = 4.4e − 09) and PDCD1LG2 (r = 0.2; P = 0.0091; Fig. 5B, C). The difference in immunotherapy response was further characterized by more immunotherapeutic genes, from which most targets were significantly upregulated in subjects with higher m6Ascores (Fig. 5D). The results strongly supported that m6Ascore was latently related to immunotherapies, and it may further predict prognosis accordingly.

### Correlation of m6Ascore and tumor mutation burden

**Fig. 6:**
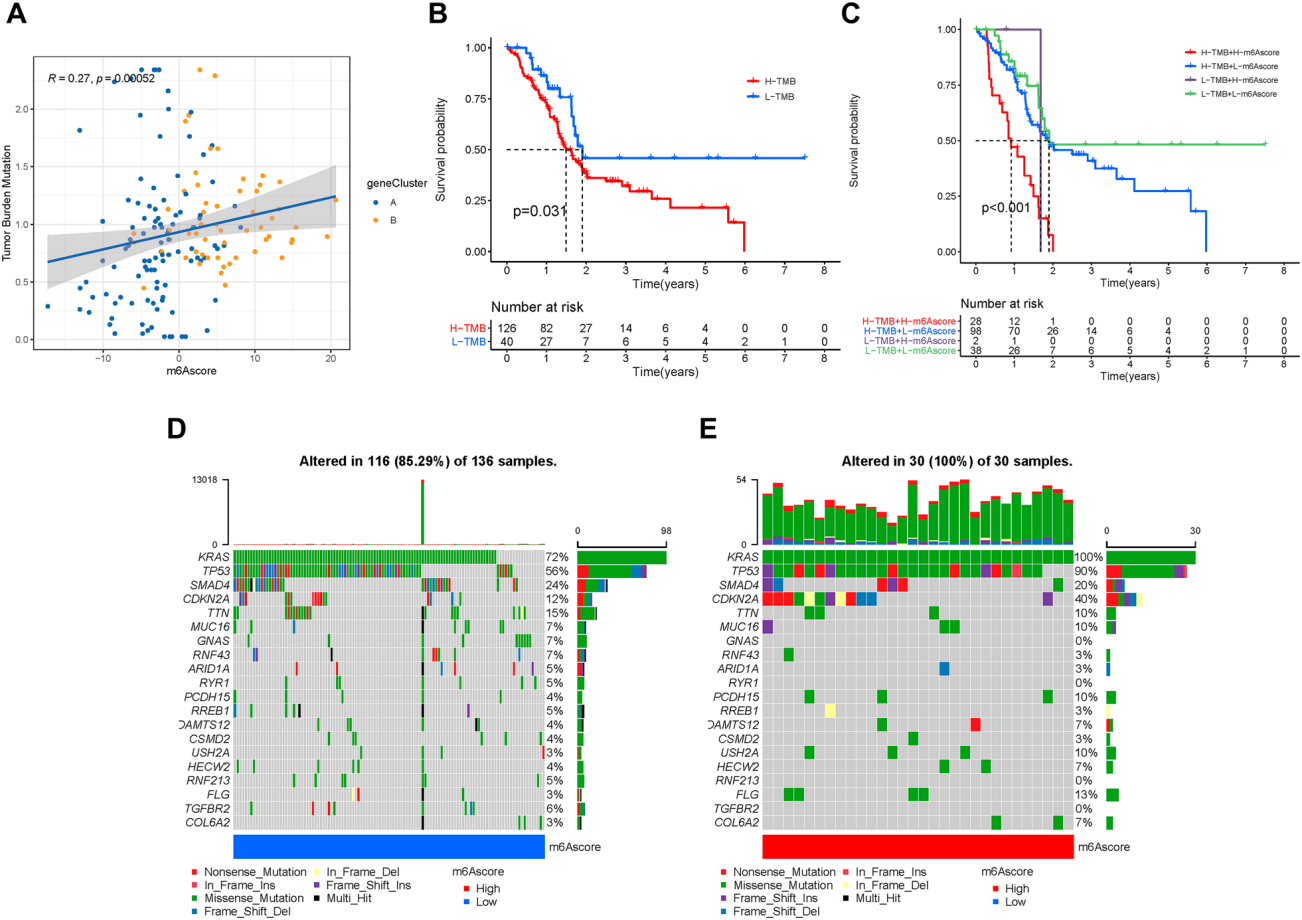
Characteristics of m6A modification patterns in tumor somatic mutation. **A** Scatterplots depicting the correlation between risk scores and TMB, P < 0.001. **B** Kaplan–Meier curves for high and low TMB groups. **C** Kaplan–Meier curves for patients stratified by both TMB and m6Ascore. The oncoPrint was constructed using **D** low m6Ascore and **F** high m6Ascore

Somatic mutations are important factors in tumor development. We investigated the correlation between the m6Ascore and TMB, and we observed that the m6Ascore was positively and significantly correlated with TMB in PAAD (Fig. 6A). Furthermore, according to the distribution patterns of TMB (Additional file 3: Fig. S3C), there was no significant difference in the mutation frequency between the low- and high-m6Ascore subgroup. Moreover, the survival curve indicated that high TMB levels were associated with a poor overall survival rate (P < 0.05, Fig. 6B). We found no interaction between m6Ascore and TMB status in terms of prognostic predictive value according to the stratified survival curve. The m6Ascore subgroups presented prominent differences in prognosis in the high and low TMB value subgroups (P < 0.001; Fig. 6C). Subsequently, we explored the significantly mutated gene (SMG) in the two m6Ascore subgroups.

According to the SMG mutational landscapes, *KRAS* (100% vs. 72%) and *TP53* (90% vs. 56%) presented higher somatic mutation rates in high m6Acore samples, and *SMAD4* (24% vs. 20%) and *TTN* (15% vs. 10%) exhibited higher somatic mutation rates in low m6Ascore samples (Fig. 6D, E). These results confirmed the impact of m6Ascore classification on genomic variation, and the potentially complex interaction between individual somatic mutations and m6A modifications.

### *TNFRSF21* remarkably correlates with prognosis and immune infiltration of PAAD

**Fig. 7:**
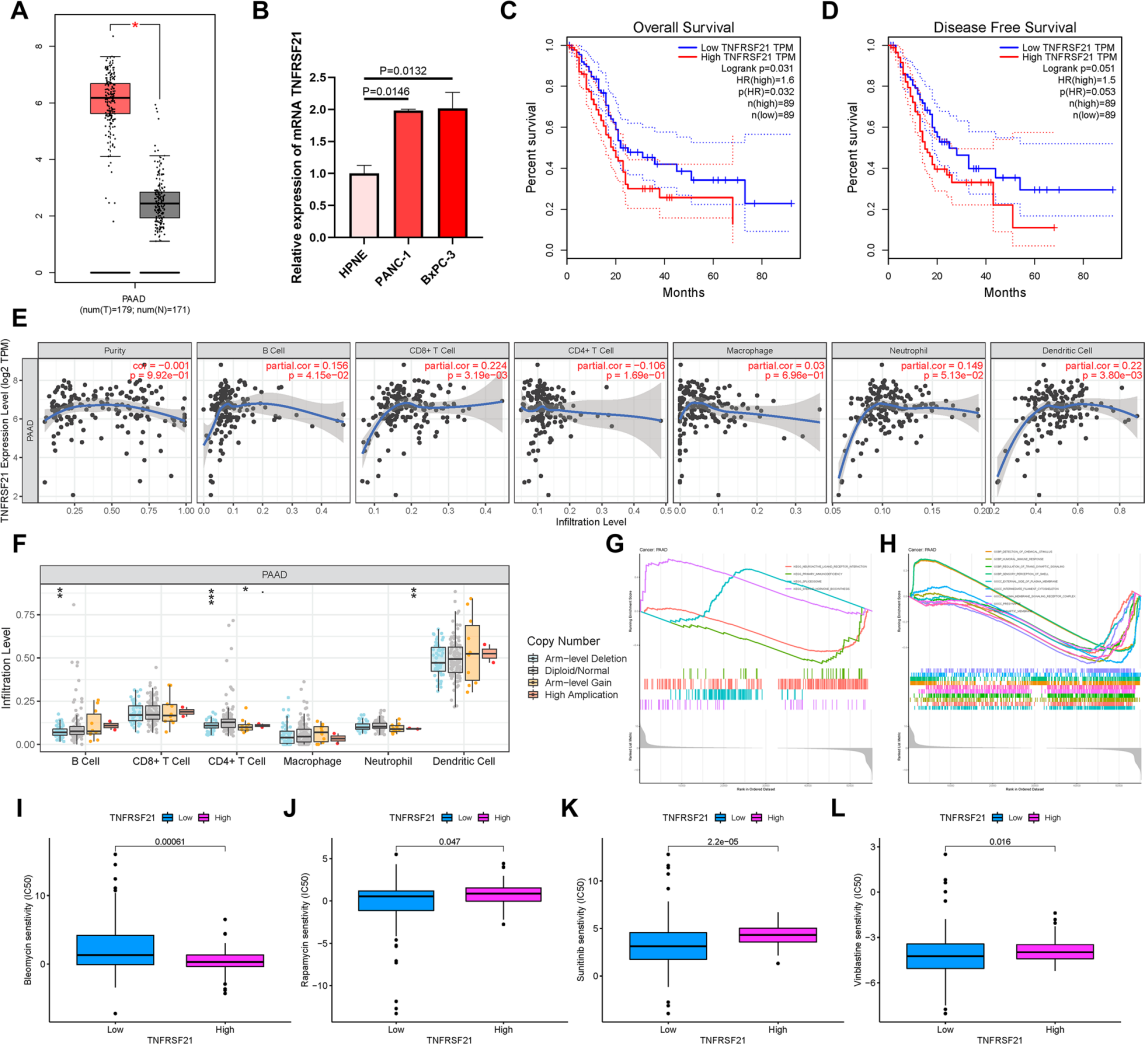
The role of in prognostic prediction, TIME, and biological processes of PAAD. **A** TNFRSF21 are upregulated in PAAD samples based on TCGA dataset. **B** TNFRSF21 are upregulated in PAAD cell lines. A lower TNFRSF21 expression level was significantly correlated with longer overall survival time **C** and disease-free survival time (**D**). **E** Correlation analysis of TNFRSF21 expression level with infiltrating B, CD4 + T, CD8 + T, dendritic cells, macrophage and neutrophil cells using TIMER. **F** Comparison of tumor infiltration levels among PAAD samples with different somatic copy number alterations in TNFRSF21. **G** Top 4 pathways enriched in the KEGG analysis in PAAD. **H** Top 5 pathways enriched in the GO analysis in PAAD. The IC50 differences of (**I**) bleomycin, (**J**) rapamycin, (**K**) sunitinib and (**L**) Vinblastine predicted by the pRRophetic algorithm between high-/low- TNFRSF21 expression subgroups

To better reveal the potential role of targets from differentially expressed genes between m6A gene clusters A and B (Fig. 3D), we analyzed *TNFRSF21* in PAAD, which has not been previously reported. Using TCGA database, the expression level of *TNFRSF21* in PAAD tumor tissues and normal pancreatic samples was analyzed. Furthermore, expression condition of *TNFRSF21* was validated using the GEPIA website [36]. The results showed that *TNFRSF21* was significantly upregulated in tumors compared with its normal counterpart (Fig. 7A). We further found that *TNFRSF21* was prominently upregulated in PAAD cell lines, such as PANC-1 and BxPC-3, compared to the normal pancreatic cell line HPNE (Fig. 7B). To explore the effect of *TNFRSF21* on survival and prognosis, survival curve analysis was performed for high- and low-expression of *TNFRSF21* in PAAD patients. It was revealed that the lower *TNFRSF21* expression presented a higher OS probability (Fig. 7C), longer disease-free survival time (Fig. 7D), and had a prominent advantage of survival. Regarding the relationship between *TNFRSF21* and immune infiltration, the results indicated that *TNFRSF21* was consistently correlated with B lymphocytes (r = 0.156; P = 4.15e − 02), CD8 + T lymphocytes (r = 0.224; P = 3.19e − 03), CD4 + T lymphocytes (r = − 0.106; P = 1.96e − 01), macrophages (r = 0.03; P = 6.96e − 01), neutrophils (r = 0.149; P = 5.13e − 02), and dendritic cells (r = 0.22; P = 3.80e − 03; Fig. 7E). Moreover, the *TNFRSF21* mutation was correlated with the infiltration of B lymphocytes, CD4 + T lymphocytes, and neutrophils (Fig. 7F).

We performed GSEA for PAAD to further explore the molecular mechanisms of *TNFRSF21* regulation in tumors. KEGG results indicated that spliceosome and steroid hormone biosynthesis were positively correlated with *TNFRSF2*1, whereas neuroactive ligand receptor interaction and primary immunodeficiency were negatively associated with *TNFRSF21* (Fig. 7G). The GO analysis showed the most significant terms that included detection of chemical stimulation, humoral immune response, regulation of trans-synaptic signaling, sensory perception of smell, and external side of the plasma membrane (Fig. 7H).

Considering the positive relationship between *TNFRSF21* and the detection of chemical stimulation and humoral immune response, we evaluated its clinical therapeutic value with chemotherapy and immune checkpoint therapy in PAAD. We found that patients with low *TNFRSF21* expression were more sensitive to bleomycin, whereas rapamycin, sunitinib, and vinblastine were more effective in patients with high *TNFRSF21* expression (Fig. 7I–L). However, as shown in Additional file 6: Fig. S6A–D, there was no significant difference in the IPS–CTLA4 and PD1/PDL1/PDL2 blocker scores between the low- and high-expression levels of *TNFRSF21* in samples.

## Discussion

Many studies have suggested that m6A modification is involved in cancer pathogenesis, progression, immunity, and inflammation, which is reflected in various TIME and tumor-related genes. Numerous studies have shown that m6A plays a dual role in several tumors [26, 37–39]. However, most studies have focused on the modulation of m6A regulators, and the comprehensive landscape of TIME, which is mediated by the complex modification of m6A, has not been systematically recognized in PAAD. Therefore, it is important to appreciate the m6A modification patterns in the characterization of TIME to further understand not only the impact of m6A modification in anti-tumor immunological regulation but also to facilitate the methods of effective precision immunotherapy.

In this study, we found that several m6A regulators were differentially expressed in tumor tissues and adjacent tissues of PAAD. Moreover, the expression of some m6A regulators was found to significantly affect the survival rate of patients with PAAD. Modification of m6A has a considerable impact on PAAD progression. To further explore the function of m6A modification in anti-tumor immunological regulation, we identified two different m6A modification patterns that corresponded to different immune phenotypes and had diverse anti-cancer immunity. The m6Acluster A could be considered as an immune-inflamed phenotype with abundant activated lymphocytes, which is associated with increased anti-tumor immunity due to high expression of immune cell‐related RNA [40]. Therefore, m6Acluster A has a better prognosis. In contrast, m6Acluster B was characterized by innate immune cell infiltration, but it presented enrichment stromal pathways and pathways markedly related to the activation of carcinogens, such as the TGF-β, Notch signaling, Wnt-β-catenin, and PI3K-AKT-mTOR signaling pathways. Abundant stromal elements prevent immune cells from recognizing and eliminating tumor cells. Therefore, m6Acluster B corresponded to an immune-excluded phenotype, for which inhibition of these stromal elements or pathways may restore anti-tumor immunity and play a positive role in PAAD therapy. For instance, previous studies have revealed that the permeation of lymphocyte cells into the parenchyma of tumors could be arrested through the activation of TGF-β- and EMT-related signaling pathways [41]. Specific TGF-β inhibitors have been identified to restore the TIME and reactivate anti-tumor immunity [42, 43]. Therefore, treatment with TGF-β blockers may be beneficial for PAAD patients with m6Acluster B.

DEGs between the two different m6A modification patterns were remarkably related to physiological processes involved in chromatin modification and signaling pathways, which proved that the mRNA transcriptome differences could be regarded as m6A related gene signatures. To better investigate the underlying molecular mechanisms, we obtained two transcriptomic subgroups based on m6A phenotype-related signature genes, similar to the clustering of m6A modification patterns.

The two m6A gene signature subgroups also possessed various clinicopathologic, prognosis, and TIME characteristics of PAAD, including biological processes and carcinogenic and stromal pathways.

Furthermore, we constructed a scoring system, named as m6Ascore, to quantify different m6A modification patterns for directing exact individual immunotherapy because of the high heterogeneity of m6A modification between individual patients. We found that the system of m6Ascore was closely related to clinical features and was confirmed to be an independent prognostic predictor of PAAD. The m6Acluster B which was regarded as an immune-excluded phenotype, had a higher m6Sig score compared to the m6Acluster A, and the high m6Sig score subgroup had a poor prognosis. The results of infiltrating immune cells showed that the m6Ascore was negatively associated with the abundance of anti-cancer immune cells, such as CD8 + T lymphocytes and B lymphocytes, which was consistent with the features of the above two m6A modification patterns [44]. Increasing evidence has revealed that TIME serves as a critical regulator of tumor progression and immunotherapeutic outcomes [45]. In fact, the m6Ascore system was efficient in mapping the diversity of the TIME and evaluating the effect of immunotherapy. We used immunotherapeutic hub targets to confirm the predictive validity of the m6Ascore. The results indicated that the m6Ascore was positively correlated with most immunotherapeutic target genes (e.g., *CD274* and *PDCD1LG2*). Therefore, the above results strongly recommend that the m6Ascore is latently related to immunotherapies and that the m6A modification pattern may contribute to the identification of immune phenotypes and decision-making in therapeutic practice.

Research on somatic mutations during tumor progression is an important basis for diagnosis, treatment, and prognostic prediction. Several studies have confirmed the close correlation between somatic mutations in the tumor genome and responsiveness to immunotherapy [46, 47]. This study found that m6Ascore was positively and significantly correlated with TMB in PAAD. Indeed, a low m6Sig score and low TMB level showed the best prognosis in PAAD. Furthermore, SMG analysis confirmed that *KRAS* and *TP53* had higher rates of somatic mutations in the group with a high m6Acore. *KRAS* and *TP53* are the most frequently mutated genes in several types of tumors, and *TP53* was found to downregulate the immunotherapeutic response in tumors [48]. In the low m6Ascore subgroup, *SMAD4* and *TTN* showed higher somatic mutation rates. *SMAD4* is involved in the TGF-β signaling pathway and generally prevents the activation of immunity in the TIME of tumors [49]. Moreover, recent studies have shown that the mutation frequency of *TTN* increases in the high immune group, which possesses abundant activated immunity in colon cancer [50], bladder cancer [51], cutaneous melanoma [52], etc. These m6Ascore-associated mutated driver genes were remarkably related to anti-tumor immune reactions and highlighted the complex interrelation of m6A modification with gene mutations in somatic cells and tumor immunogenomic regulation. This is helpful to better understand the complexity of the TIME.

TNFRSF21, also known as death receptor 6, is a member of the tumor necrosis factor receptor superfamily [53, 54]. TNFRSF21 is widely expressed in several types of tissues and various cultured cells [53], and it has been reported that TNFRSF21 induces apoptosis in some tumors [55, 56]. The present study reported that NF‑κB could protect the survival signaling of TNFRSF21‑induced apoptosis by regulating the expression of TNFRSF21 [57]. MiR‑20a‑5p targets TNFRSF21 to downregulate its expression, thus promoting cell proliferation, migration, and invasion capacities in head and neck squamous cell carcinoma [58]. However, the molecular function and carcinogenesis of TNFRSF21 in PAAD are not well understood. Our research showed that TNFRSF21 was significantly overexpressed in PAAD and that high expression of TNFRSF21 was correlated with poor prognosis of PAAD in patients. Furthermore, TNFRSF21 was positively associated with immune infiltration by B lymphocytes, CD8 + T lymphocytes, dendritic cells, and macrophages. In addition, the risk scoring scheme revealed that sensitivity to chemotherapy drugs was associated with TNFRSF21 expression levels. Therefore, PAAD patients might be more suitable for distinct combination administration of molecular targeting and chemotherapeutic agents according to TNFRSF21 stratification. However, the biological role of TNFRSF21 in PAAD remains unknown and requires further experimental exploration. In our study, we systematically identified different m6A methylation modification patterns among 176 samples of PAAD patients based on 23 m6A regulators. Furthermore, we comprehensively analyzed the complexity and heterogeneity of individual TIME utilizing distinct m6A modification patterns, which is an important basis for the regulation of anti-tumor immunity. The system of m6Ascore was constructed as an independent prognostic predictor of PAAD. The m6Ascore was also confirmed by mapping the diversity of the TIME and evaluating the effect of immunotherapy, which may be beneficial for patients with PAAD. Moreover, we confirmed the complicated relationship and cooperative effect between m6Ascore and TMB. In summary, the comprehensive assessment of m6A modification patterns in PAAD will provide novel insights into the TIME landscape and guide accurate immunotherapy of individuals.

## Supplementary Information

**Additional file 1: Figure S1.** The survival curves of the m6A regulators were estimated by the Kaplan–Meier plotter.

**Additional file 2: Figure S2.** Consensus clustering based on the m6A modification patterns. (A**–**D) Consensus matrixes of PAAD samples for each k (k = 2–5), displaying the clustering stability using 1000 iterations of hierarchical clustering.

**Additional file 3: Figure S3.** (A) 169 m6A-related differentially expressed genes (DEGs) between two m6A-clusters are shown in the Venn diagram. (B) Difference in m6Ascore between subgroups according to clinical stage. (C) Differences in TMB between patients in the low-and high-risk score subgroups.

**Additional file 4: Figure S4**. Consensus clustering based on DEGs of different m6A modification clusters. (A**–**D) Consensus matrixes of HCC cohorts for each k (k = 2–5), displaying the clustering stability using 1000 iterations of hierarchical clustering.

**Additional file 5: Figure S5.** Kaplan–Meier survival analysis for multiple HCC subgroups stratified by clinical variables. (A**–**B) Age. (C**–**D) Gender. (E–F) Stage.

**Additional file 6: Figure S6.** Immunotherapeutic predictive significance of TNFRSF21 in PAAD. (A) IPS score distribution plot. (B) IPS–CTLA4 blocker score distribution plot. (C) IPS–PD1/PDL1/PDL2 blocker score distribution plot. (D) IPS–CTLA4 and PD1/PDL1/PDL2 blocker score distribution plot.

**Additional file 7. **Additional Tables.

## Data Availability

The datasets generated for this study are available in TCGA database (https://portal.gdc.cancer.gov) and GDC (https://portal.gdc.cancer.gov/).
